# Dynamic immune signatures of patients with advanced non–small-cell lung cancer for infection prediction after immunotherapy

**DOI:** 10.3389/fimmu.2024.1269253

**Published:** 2024-01-26

**Authors:** Yung-Hung Luo, Chia-I Shen, Chi-Lu Chiang, Hsu-Ching Huang, Yuh-Min Chen

**Affiliations:** ^1^ Department of Chest Medicine, Taipei Veterans General Hospital, Taipei, Taiwan; ^2^ School of Medicine, College of Medicine, National Yang Ming Chiao Tung University, Taipei, Taiwan; ^3^ Institute of Clinical Medicine, College of Medicine, National Yang Ming Chiao Tung University, Taipei, Taiwan

**Keywords:** lung cancer, pulmonary infection, immunotherapy, immune signature, cytokines

## Abstract

**Background:**

Pulmonary infections are a crucial health concern for patients with advanced non–small-cell lung cancer (NSCLC). Whether the clinical outcome of pulmonary infection is influenced by immunotherapy(IO) remains unclear. By evaluating immune signatures, this study investigated the post-immunotherapy risk of pulmonary infection in patients with lung cancer and identified circulating biomarkers that predict post-immunotherapy infection.

**Methods:**

Blood specimens were prospectively collected from patients with NSCLC before and after chemotherapy(C/T) and/or IO to explore dynamic changes in immune signatures. Real-world clinical data were extracted from medical records for outcome evaluation. Mass cytometry and ELISA were employed to analyze immune signatures and cytokine profiles to reveal potential correlations between immune profiles and the risk of infection.

**Results:**

The retrospective cohort included 283 patients with advanced NSCLC. IO was associated with a lower risk of pneumonia (odds ratio=0.46, p=0.012). Patients receiving IO and remained pneumonia-free exhibited the most favorable survival outcomes compared with those who received C/T or developed pneumonia (p<0.001). The prospective cohort enrolled 30 patients. The proportion of circulating NK cells significantly increased after treatment in IO alone (p<0.001) and C/T+IO group (p<0.01). An increase in cell densities of circulating PD-1^+^CD8^+^(cytotoxic) T cells (p<0.01) and PD-1^+^CD4^+^ T cells (p<0.01) were observed in C/T alone group after treatment. In IO alone group, a decrease in cell densities of TIM-3^+^ and PD-1^+^ cytotoxic T cells (p<0.05), and PD-1^+^CD4^+^ T cells (p<0.01) were observed after treatment. In C/T alone and C/T+IO groups, cell densities of circulating PD-1^+^ cytotoxic T cells significantly increased in patients with pneumonia after treatment(p<0.05). However, in IO alone group, cell density of PD-1^+^ cytotoxic T cells significantly decreased in patients without pneumonia after treatment (p<0.05). TNF-α significantly increased after treatment with IO alone (p<0.05) but decreased after C/T alone (p<0.01).

**Conclusions:**

Our results indicate that the incorporation of immunotherapy into treatment regimens may potentially offer protective effects against pulmonary infection. Protective effects are associated with reduction of exhausted T-cells and augmentation of TNF-α and NK cells. Exhausted T cells, NK cells, and TNF-α may play crucial roles in immune responses against infections. These observations highlight the potential utility of certain circulating biomarkers, particularly exhausted T cells, for predicting post-treatment infections.

## 1 Introduction

Lung cancer is the leading cause of malignancy-related fatalities globally, including in Taiwan. Non–small-cell lung cancer (NSCLC) is the most common type of lung cancer, with more than half of the affected patients presenting with locally advanced or metastatic disease at diagnosis (1, 2). The high metastatic potential, pervasive development of drug resistance, and frequent recurrence observed in advanced lung cancer contribute to its low survival rates. Immunotherapy has emerged as an effective treatment option and has been demonstrated to improve patient outcomes (3–10). Cancer immune evasion is associated with tumor-extrinsic mechanisms that lead to an immunosuppressive tumor microenvironment that enables tumors to evade immune surveillance (11–16). The existence of exhausted PD-1^+^ T cells was strongly predictive of treatment response and survival time in a study of NSCLC patients treated with PD-1 checkpoint inhibitors (17). TIM-3 expression on T cells has been related to nodal metastasis and advanced lung cancer stages (18). The expression of LAG-3 on tumor-infiltrating lymphocytes has demonstrated the correlation with early postoperative recurrence and poor prognosis in patients with NSCLC (19). Immune checkpoint inhibitors (ICIs) have emerged as a useful tool to enable the human immune system to target and eliminate tumor cells (8, 20). However, the modulation of the immune system through immunotherapy can exert both positive and negative effects (21, 22). With the expanding use of ICIs, our collective experience has revealed a range of immunotherapy-related adverse events (irAEs) such as dermatitis, pneumonitis, and endocrine dysfunction (23). Treatment of irAEs involves the use of steroids and immunosuppressants, which may increase the risk of infection (24, 25). Both the dysfunction of T cells and the application of immunosuppressants have been linked to increased susceptibility to infections (21, 22). An inhibitory role of PD-1, TIM-3, and LAG-3 in T cell responses has been reported (26–28), the upregulation of PD-1, TIM-3, LAG-3 in exhausted T cells has been found in both chronic infection and tumors (18, 27–30). Enhanced T cell apoptosis and PD-1 upregulation on CD8+ T cells were found in patients with chronic infection (31). Upregulation of TIM-3 on exhausted CD8^+^ and CD4^+^ T cells upon infection by human immunodeficiency virus type 1 has been reported (32). Increased LAG-3 expression was reported to suppress T-cell function in chronic hepatitis B. Previous studies have demonstrated that LAG-3 can suppress the CD8^+^ T-cell response in chronic viral infection (33). Collectively, T cell exhaustion has been shown to correlate with impaired immunity against chronic infections and lung cancer.

Pulmonary infections, like pneumonia, influenza, and other emerging infectious diseases, have been reported to be more fatal in patients with lung cancer compared to the general population (34, 35). Infections can also interrupt the continuity of systemic cancer treatment. To improve the survival rates of patients with cancer, clinical physicians must be increasingly vigilant about monitoring and managing infections, as these profoundly impact overall survival outcomes (21, 22, 35). Previous clinical trial data have not consistently demonstrated a significantly higher risk of infection in the ICI treatment group compared to the chemotherapy group (36–38). However, some studies reveal conflicting evidence, suggesting a potential link between the risk of cancer and infection (21, 34). Factors such as the administration of steroids and the presence of underlying comorbidities may play an important role in post-ICI infection (25). Taken together, there have been limited reports on the impact of different anti-cancer therapies, including ICIs, on the immunity against pathogens. Moreover, whether the use of ICIs influences the incidence of infections remains unclear (22).

Mass cytometry (cytometry by time-of-flight [CyTOF]) is a next-generation cytometry platform incorporating numerous technological advances, offering distinct advantages over conventional fluorescence-based flow cytometry (39). Studies utilizing CyTOF have reported the implications of exhausted T cells in lung cancer patients, including their associations with disease progression and responses to immunotherapy (40–42). Although emerging evidence highlights the interplay between immune cells and immunotherapy, the exact underlying mechanisms remain to be fully elucidated. CyTOF has also been applied to investigate immune alterations in infectious diseases such as human immunodeficiency virus, influenza, and sepsis (43, 44), enabling comprehensive analysis of various immune cell populations simultaneously. In our current study, CyTOF was used in one prospective cohort to evaluate immune cell phenotypes in patients with lung cancer receiving ICI treatment. Additionally, real-world clinical data were collected in a separate retrospective cohort to assess the clinical outcomes. By evaluating clinical manifestations and immune signatures, this study consisting of a prospective cohort and a retrospective cohort investigated the risk of infections following immunotherapy for lung cancer and examined circulating biomarkers that could serve as predictors of infections following immunotherapy.

## 2 Patients and methods

### 2.1 Patient population

This study consisted of two cohorts, including a retrospective cohort and a prospective cohort. For the retrospective cohort, we collected real-world medical records from Taipei Veterans General Hospital, a tertiary medical center in Taiwan. Patients with stage IV NSCLC who harbored wild-type *EGFR* and *ALK* treated with either chemotherapy alone, immunotherapy alone, or both were included. The immunotherapy included PD-1/PD-L1 inhibitors, and the chemotherapy consisted of platinum-doublet chemotherapy and single-agent chemotherapy according to the clinical practice guidelines (45). The following patients were excluded from the analysis: (1) those with known *EGFR* or *ALK* mutations who had received first-line targeted therapy; (2) those diagnosed with small-cell carcinoma; and (3) those who underwent double immunotherapy. The period of patient enrollment spanned from January 1, 2018, to December 31, 2020, while the data collection and follow-up time for the monitoring of the infection episodes was from January 1, 2018, to March 31, 2022. The study was approved by the Institutional Review Board of Taipei Veterans General Hospital (2022-08-010BC), and the requirement for informed consent was waived.

For the prospective cohort, patients with NSCLC were recruited from Taipei Veterans General Hospital from 2020 to 2022. Patients with stage IV NSCLC undergoing systemic treatment, such as immunotherapy (IO), chemotherapy (C/T), or both were included. Patients were categorized into three groups based on their treatment modalities: the C/T alone group, the IO alone group, and the IO+C/T group. The C/T alone group received chemotherapy only, the IO alone group received immunotherapy only, and the IO+C/T group received a combination of immunotherapy and chemotherapy. The following patients were excluded from this study: (1) patients who had received steroids at a dosage exceeding prednisolone 10 mg/day within the preceding 14 days and (2) patients who had undergone major surgery or radiotherapy within the preceding 30 days; (3) patients with known *EGFR* or *ALK* mutation, received first-line targeted therapy; (4) patients diagnosed with small-cell carcinoma; (5) patients who underwent double immunotherapy. Medical records were reviewed to collect crucial clinical characteristics, including age, sex, tumor node metastasis (TNM) staging, systemic treatment regimen, and the timing of infection. The blood specimens were collected for the immunophenotyping analysis. The study was approved by the Institutional Review Board of Taipei Veterans General Hospital (2020-05-006B), and all participants provided informed consent prior to participation in this study.

### 2.2 Specimen collection

Whole-blood specimens were collected through phlebotomy from the patients on the day before starting systemic treatment (Day 0) and on one week after receiving first dose of medication (Day 8). Peripheral blood mononuclear cells (PBMCs) were isolated by Ficoll density gradient centrifugation within 6 hours from the time of blood collection. The isolated PBMCs were cryopreserved until subsequent testing and analysis.

### 2.3 Mass cytometry

The immunophenotyping of various lymphocyte subpopulations and monocytes were performed by mass cytometry (CyTOF). The experiment started with a careful design of the antibody/probe panel. This was followed by sample analyzing by CyTOF 2 mass cytometer (Fluidigm, San Francisco, CA, USA), uploading the flow cytometry standard (FCS) file to the online FCS file-processing platforms, and finally data analysis (**Supplementary Figure S1**). The CyTOF staining process involved several sequential steps. Initially, the cells were treated with a rhodium viability staining reagent (Fluidigm) for 10 min before commencing the staining procedure. Subsequently, Cell-ID Intercalator-103Rh (Fluidigm, Catalog #201103A) was added to the cells, with incubation for 10 minutes before the introduction of CyTOF staining antibodies. The dilution of antibodies started at the concentration recommended by the manufacturer (1 μl antibody per 100 μl cell suspension containing 3 × 10^6^ cells). According to the manufacturer’s protocol for staining the antibody, 50 μL of the 2X antibody cocktail was added to each tube and the total staining volume is 100 μL (50 μL of cell suspension + 50 μL 2X antibody cocktail). Then, the cells were incubated with intracellular antibodies for 30 minutes. Following incubation, the cells were washed once with CyTOF staining buffer (a solution containing cell staining buffer, calcium/magnesium-free PBS, 0.2% BSA, and 0.05% sodium azide; Fluidigm; Catalog #201068, San Francisco, CA, USA). To fix the cells, 1.6% formaldehyde buffer (16% formaldehyde [weight by volume], methanol-free dilution; Thermo Scientific™, Catalog #28908, Waltham, MA, USA) was used for 10 minutes. After fixation, the cells were washed twice with CyTOF staining buffer. Finally, the cells were diluted in distilled water following the last washing step and then injected into the mass cytometer. These cells were subsequently analyzed using a CyTOF 2 mass cytometer (Fluidigm) in accordance with the manufacturer’s guidelines. Data analysis was performed using OIMQ data analysis software (OMIQ, Inc., Santa Clara, CA, USA). Lastly, the gated CD45^+^ cell population was clustered based on all labeled phenotypic markers through the spanning-tree progression analysis of density-normalized events (SPADE) method (OMIQ, Inc. Santa Clara, CA, USA).

### 2.4 Immunophenotyping through mass cytometry

The immunophenotyping of the following lymphocyte and monocyte subpopulations was conducted through CyTOF (Fluidigm) under various conditions: T cells (CD45^+^CD3^+^) labeled with antibodies of Catalog #3141009C and #3170007C; cytotoxic T cells (CD45^+^CD3^+^CD8^+^) labeled with antibodies of Catalog #3141009C, #3170007C, and #3146001C; helper T cells (CD45^+^CD3^+^CD4^+^) labeled with antibodies of Catalog #3141009C, #3170007C and #3145001B; natural killer (NK) cells (CD45^+^CD16^+^) labeled with antibodies of Catalog #3141009C and #3209002C; exhausted T cells (CD45^+^CD3^+^CD8^+^PD1^+^ or CD45^+^CD3^+^CD8^+^TIM3^+^ or CD45^+^CD3^+^CD8^+^LAG3^+^) labeled with antibodies of Catalog #3141009C, #3170007C, #3146001C, #3155009C, #3153008C, and #3165037C; B cells (CD45^+^CD19^+^) labeled with antibodies of Catalog #3141009C, and #3158032C; and monocytes (CD45^+^HLADR^+^CD14^+^) labeled with antibodies of Catalog #3141009C, #3174001C, and #3160006B (all from Fluidigm) (46–50). To manage the multidimensional data obtained from CyTOF, we used specialized tools in the OIMQ data analysis software (OMIQ, Inc.), including the optimized t-Distributed Stochastic Neighbor Embedding (opt-SNE) plot and histogram, which facilitated dimensionality reduction and offered a comprehensive visualization of events within a 2-dimensional map derived from the multidimensional data (51). Immune signatures in PBMCs from healthy controls (n = 3) were analyzed through mass cytometry (CyTOF), and the opt-SNE plots and histograms revealed the obvious expression of CyTOF markers, including CD45, CD3, CD8, CD4, CD16, PD-1, TIM-3, and LAG-3, in the PBMCs (**Supplementary Figure S2**).

### 2.5 Enzyme-linked immunosorbent assay of cytokine levels

The blood specimens collected from the various treatment groups were centrifuged at 4°C and 1500 rpm. The resulting plasma was collected and cryopreserved until further testing. To determine the concentrations of various proteins in the plasma, we employed the Quantikine enzyme-linked immunosorbent assay (ELISA) Kit (R&D Systems, Minneapolis, MN, USA; Human interleukin [IL]-2 ELISA Kit, Catalog #D2050; Human IL-6 ELISA Kit, Catalog #D6050; Human IL-10 ELISA Kit, Catalog #D1000B; Human tumor necrosis factor-alpha [TNF-α] ELISA Kit, Catalog #DTA00D; Human interferon-gamma [IFN-γ] ELISA Kit, Catalog #DIF50C). According to the manufacturers’ protocol, the processed specimens were incubated at room temperature for 2 hours, after which they were subjected to four washes with the given wash buffer. The recommended volume of conjugate antibody was added to each well. The washing step was then repeated, after which 200 μL of substrate solution was added to each well, and the processed specimens were incubated at room temperature for 30 minutes. Then, 50 μL of stop solution was added to each well. The experimental plates were read on a Spectramax iD3 plate reader (Molecular Devices, San Jose, CA, USA) at 450 nm within 30 minutes. Positive and negative controls were included in the analysis. The change of cytokine levels was demonstrated by using Log_2_ fold change in the concentration of different cytokines in Day 8 (D8) compared with that in Day 0 (D0) (52).

### 2.6 Outcome evaluation

Clinical data, including age, sex, smoking history, performance status, comorbidities, and the lines of cancer treatment, were collected and analyzed. Radiotherapies, both in the thoracic and extrathoracic areas, were recorded. Neutropenia, defined as an absolute neutrophil count (ANC) of <500/mm^3^, was recorded. IrAEs were recorded based on the review of medical records. The diagnosis of infections or pneumonia in the patients with lung cancer was made by clinical physicians in adherence to established clinical practice guidelines (53–55). A comprehensive evaluation following practice guidelines was conducted to differentiate between infectious pneumonia and pneumonitis related to immunotherapy or radiation. The evaluation involved assessing symptoms, performing chest CT scans, bronchoscopy with bronchoalveolar lavage (BAL), evaluating pulmonary function, conducting lung biopsy and determining the appropriate therapy (56–58). In the retrospective cohort, steroid exposure was defined as the daily administration of prednisone or other equivalent steroids at a dose of 10 mg or higher for at least 10 days. Infection episodes requiring the administration of either oral or intravenous antibiotics were identified through a review of medical records. The types of infection included (1) pneumonia, (2) urinary tract infection, (3) bacteremia, (4) skin and soft tissue infection, and (5) others (including intraabdominal infection, colitis, neutropenic fever, and occult infection). The infection episodes were counted, and any instances that necessitated hospitalization or admission to the intensive care unit (ICU) were also documented. Treatment response evaluation was performed according to the Response Evaluation Criteria in Solid Tumors (RECIST) group criteria (version 1.1). Progression-free survival (PFS) was calculated from the date of treatment commencement to the earliest identified sign of disease progression, as determined by the RECIST criteria, or the date of death from any cause. If disease progression had not occurred at the last follow-up visit, PFS was considered censored at that time point. Furthermore, overall survival (OS) was computed from the date of treatment commencement until the date of death or last follow-up visit. Throughout the follow-up period, any incidence of pneumonia/irAEs was also recorded.

### 2.7 Statistical analysis

Categorical variables were assessed using Pearson’s chi-squared test or Fisher’s exact test. Continuous variables were compared using Student’s *t* test and the Mann–Whitney U test. To assess the risk factors for infection and pneumonia, logistic regression models were used. Survival analysis was conducted by using the Kaplan–Meier method with a log-rank test. The Cox proportional-hazards regression model was applied for univariate and multivariate survival analyses. Variables that exhibited a significance level of p < 0.1 in the univariate analysis were included in the multivariate analysis. Statistical significance was set at p < 0.05 (two-sided). SPSS software (version 21.0, SPSS Inc., Chicago, IL, USA) was used for all the analyses.

## 3 Results

### 3.1 Patient characteristics

In the retrospective cohort, a total of 283 patients with stage IV NSCLC were included. These patients were categorized into three groups according to their treatment regimen: C/T group (n = 139), IO group (n = 63), and C/T+IO group (n = 81). The median age of the cohort was 63 years (range, 33–96 years). Among all patients, 45.9% were male, and 37.7% were ever-smokers. The majority of the patients exhibited an Eastern Cooperative Oncology Group performance status (ECOG PS) of 0–1, and 24.3% of the patients had received more than two lines of treatment. Among the three groups, the median age was significantly higher in the IO alone group. A greater proportion of patients were male and had smoking history, ECOG PS 0–1, and radiotherapy in the IO alone group. The patients who received the C/T+IO treatment exhibited higher rates of neutropenia and previous steroid exposure. A greater proportion of patients who received C/T alone had more than two lines of treatment (**Table 1**). No difference in the frequency of comorbidities such as diabetes mellitus (DM), chronic obstructive pulmonary disease (COPD), and chronic kidney disease (CKD) was noted among the three groups. All the baseline characteristics are listed in **Table 1**.

**Table 1:** Baseline characteristics of the retrospective cohort (n = 283).

Characteristics (Patient number)	All patients (283)	C/T alone (139)	IO alone (63)	C/T+IO (81)	P value
Age (median, range)	63 (33-96)	65 (36-96)	68 (44-90)	62 (33-84)	0.024
Male (%)	191 (45.9)	77 (55.4)	55 (87.3)	59 (72.8)	<0.001
Smoking (%)	157 (37.7)	58 (41)	45 (71.4)	54 (66.7)	<0.001
ECOG PS ≥2 (%)	22 (5.3)	21 (15.1)	0 (0)	1 (1.2)	<0.001
DM	58 (13.9)	27 (19.4)	17 (27.0)	14 (17.3)	0.332
COPD	26 (6.3)	11 (7.9)	7 (11.1)	8 (9.9)	0.743
CKD	14 (3.4)	9 (6.5)	3 (4.8)	2 (2.5)	0.459
Neutropenia	26 (6.3)	4 (2.9)	0 (0.0)	22 (27.2)	<0.001
Steroid	36 (8.7)	6 (4.3)	11 (17.5)	19 (23.5)	<0.001
Treatment lines ≥2	101 (24.3)	67 (48.2)	26 (41.3)	8 (9.9)	<0.001
Radiotherapy	69 (16.6)	21 (15.1)	27 (42.9)	21 (25.9)	<0.001

C/T, chemotherapy; CKD, chronic kidney disease; COPD, chronic obstructive pulmonary disease; DM, diabetes mellitus; ECOG PS, Eastern Cooperative Oncology Group performance status; IO, immunotherapy.

### 3.2 Incidence of infection

Of the 283 patients, 170 (40.9%) had one or more episodes of infection (**Supplementary Table S1**). No significant differences in the incidence of infection were found among the three groups. Pneumonia was the most prevalent type of infection, accounting for 36% of infections and exhibiting a relatively consistent incidence across the three groups (**Supplementary Table S1**). In addition to pneumonia, the other types of infection episodes were urinary tract infection (1.0%), bacteremia (1.4%), and soft tissue infection (4.8%), with no significant differences in incidence among the three groups. Notably, a higher incidence of other infection types was observed in the C/T+IO group (22.2%) compared with the C/T alone group (7.9%) and IO alone group (14.3%) (p = 0.011; **Supplementary Table S1**). Among patients who developed infections, 36.3% required hospitalization, and 10.3% were indicated for ICU admission (**Table 2**). However, the incidence of hospitalization and ICU admission did not significantly differ among three groups (**Table 2**). Only one patient (0.4%) who received IO alone treatment had coronavirus disease 2019 (COVID-19) pneumonia.

**Table 2:** Characteristics of patients with infection in the retrospective cohort (n = 283).

Characteristics (Patient number)	All patients (283)	C/T alone (139)	IO alone (63)	C/T+IO (81)	P value
Infection (%)^a^	170 (40.9)	82 (59)	37 (58.7)	51 (63)	0.832
More than one infection (%)	74 (17.8)	36 (25.9)	14 (22.2)	24 (29.6)	0.593
Infection require hospitalization (%)	151 (36.3)	72 (51.8)	34 (54)	45 (55.6)	0.859
ICU admission due to infection (%)	43 (10.3)	25 (18)	9 (14.3)	9 (11.1)	0.387

a One patient (0.4%) with coronavirus disease 2019 pneumonia in the IO alone group.

C/T, chemotherapy; ICU, intensive care unit; IO, immunotherapy.

### 3.3 Risk factors and survival analysis of infection and pneumonia

**Figure 1:**
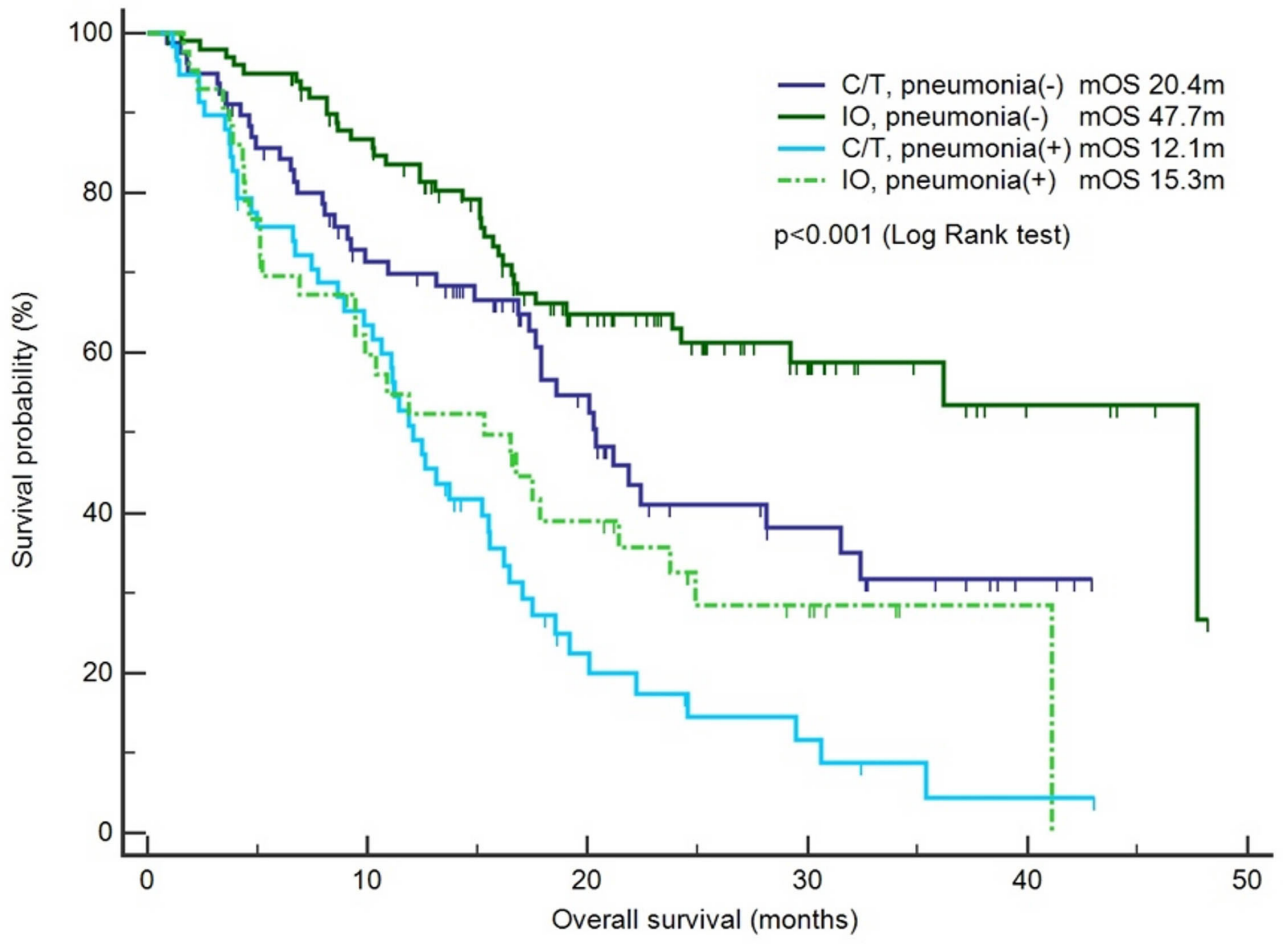
Survival outcomes between the chemotherapy and immunotherapy groups with or without pneumonia. The patients were categorized into the chemotherapy group (C/T) and immunotherapy group (IO), and a survival analysis was performed for the patients with pneumonia or without pneumonia (n = 283).

The risk factors for infection and pneumonia were analyzed using logistic regression. In the univariate analysis, male sex and CKD exhibited increased odds ratios (ORs) for infection. However, in the multivariate analysis, only CKD was significantly associated with a higher risk of infection (OR: 5.05, 95% confidence interval [CI]: 1.06–24.15, p = 0.043; **Supplementary Table S2**). Immunotherapy was not associated with the risk of infection. In the context of pneumonia incidence, comorbidities, sex, and smoking history exhibited no statistical significance (**Table 3**). However, immunotherapy was associated with a lower risk of pneumonia after adjustment for other factors (OR: 0.46, 95% CI: 0.25–0.84, p = 0.012; **Table 3**). The IO+C/T treatment was not associated with higher risk of pneumonia compared to the IO alone treatment (OR: 1.17, 95% CI: 0.57–2.40, p = 0.663; **Table 3**). In addition, immunotherapy was not related to the risk of COVID-19 pneumonia in the univariate and multivariate analysis (**Supplementary Table S3**). Furthermore, a survival analysis was performed for the patients with or without pneumonia. The patients were categorized into the chemotherapy group and immunotherapy group which consisted of IO alone group and C/T+IO group according to their exposure to immunotherapy. The patients without pneumonia exhibited more favorable OS than those with pneumonia in both the aforementioned groups. Among all groups, the patients who received immunotherapy and did not develop pneumonia exhibited the most favorable survival outcome, with a median OS of 47.7 months (95% CI: 25.9–69.5, p < 0.001; **Figure 1**). In C/T alone group, patients with pneumonia had lower OS than those without (median OS: 12.1 vs. 20.4 months, p < 0.001). In IO alone group, patients with pneumonia had lower OS than those without (median OS: 9.4 vs. not reached, p < 0.001). In C/T+IO alone group, patients with pneumonia tended to have lower OS than those without (median OS: 23.7 vs. 47.7 months, p = 0.161) (Data not shown). The patients with pneumonia other than that related to COVID-19 did not exhibit better OS than those with COVID-19 pneumonia (p = 0.325). In summary, the findings suggest that the administration of immunotherapy in patients with lung cancer not only is related to a lower risk of pneumonia but may also confers survival benefits by preventing pneumonia.

**Table 3:** Multivariate analysis of pneumonia episodes (n = 283).

	Univariate analysis			Multivariate analysis		
	OR	95% CI	p value	OR	95% CI	p value
Male	1.45	0.85-2.46	0.174	1.80	0.97-3.35	0.062
Age ≥ 70	1.04	0.63-1.71	0.895			
Smoking history	1.03	0.63-1.67	0.918	0.93	0.53-1.66	0.816
ECOG PS ≥ 2	1.02	0.41-2.51	0.974	0.72	0.28-1.86	0.494
DM	1.01	0.55-1.84	0.977			
COPD	0.93	0.40-2.18	0.874			
CKD	2.48	0.84-7.37	0.101			
Neutropenia	1.12	0.49-2.57	0.788	1.47	0.60-3.60	0.397
Steroid	0.46	0.20-1.06	0.067	0.54	0.23-1.28	0.161
Treatment lines ≥ 2	1.19	0.72-1.97	0.502	0.95	0.54-1.68	0.853
Radiotherapy	1.19	0.68-2.08	0.539	1.45	0.77-2.71	0.252
With immunotherapy^a^	0.58	0.35-0.94	0.028	0.46	0.25-0.84	0.012
With chemotherapy^b^	1.28	0.70-2.32	0.421			

a IO+C/T was not associated with higher risk of pneumonia compared with IO alone (OR: 1.17, 95% CI: 0.57-2.40, p = 0.663).

b The exposure to chemotherapy, including C/T+IO and C/T alone groups, was not associated with higher risk of pneumonia compared with IO alone.

C/T, chemotherapy; CI, confidence interval; CKD, chronic kidney disease; COPD, chronic obstructive pulmonary disease; DM, diabetes mellitus; ECOG PS, Eastern Cooperative Oncology Group performance status; IO, immunotherapy; OR, odds ratio.

### 3.4 Analysis of circulating biomarkers and immune signatures

Circulating biomarkers and immune signatures that could predict the occurrence of post-treatment pneumonia among the patients receiving various lung cancer therapies were investigated through mass cytometry (CyTOF). A total of 30 patients were enrolled in the prospective cohort, and the patients’ blood specimens were analyzed through CyTOF. The included patients were categorized into three groups based on their treatment regimens: C/T alone group (n = 6), IO alone group (n = 18) and C/T+IO group (n = 6). The patient characteristics were listed in **Supplementary Table S4**. There was no patient with COVID-19 pneumonia in the prospective cohort. In the context of pneumonia incidence, the comorbidities, age, sex, smoking history, and treatment modalities showed no statistical significance (**Supplementary Table S5**).

**Figure 2:**
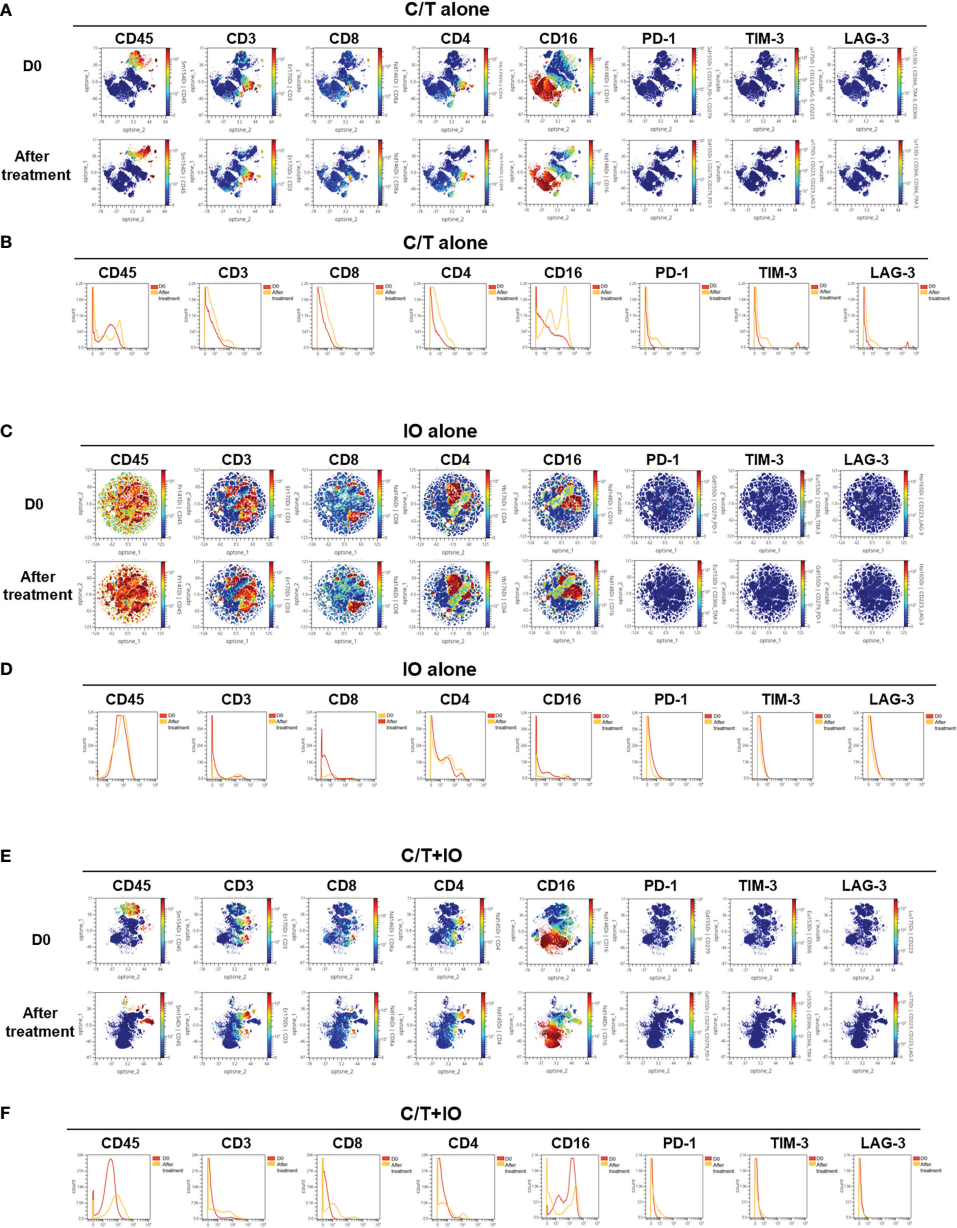
Immune signatures in the peripheral blood of patients, as analyzed through mass cytometry (CyTOF). The optimized t-Distributed Stochastic Neighbor Embedding (opt-SNE) plots **(A, C, E)** and histograms **(B, D, F)** depict the changes in lymphocyte expression in the peripheral blood mononuclear cells across the different treatment groups, including **(A, B)** chemotherapy (C/T) alone group, **(C, D)** immunotherapy (IO) alone group, and **(E, F)** C/T+IO group (n = 30).

**Figure 3:**
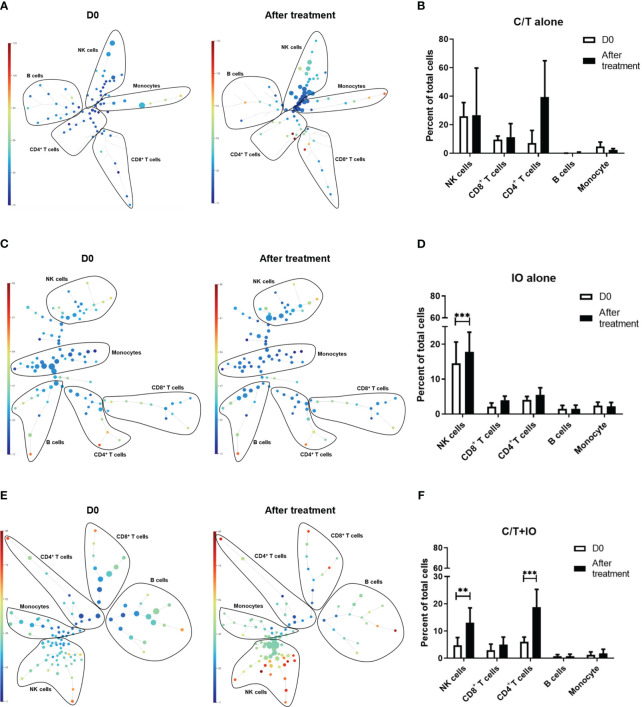
Spanning-tree progression analysis of density-normalized events (SPADE) trees visualizing the clustering and quantization of immune cell expression. The SPADE trees and bar graphs illustrate different immune cell subpopulations and proportions of various immune cell types, including NK cells, CD8^+^ (cytotoxic) T cells, CD4^+^ T cells, B cells, and monocytes, in different treatment groups before and after treatment, including **(A, B)** chemotherapy (C/T) alone group, **(C, D)** immunotherapy (IO) alone group (***p < 0.001), and **(E, F)** C/T+IO group (**p < 0.01) (n = 30).

This study employed opt-SNE plots and overlay histograms to visually depict the changes in lymphocyte number in PBMCs among different treatment groups (**Figure 2**). The spectral colors overlaid on the opt-SNE plots illustrate the marker expression patterns of the different cell populations. In the prospective cohort, ten of the thirty patients received more than two lines of treatment before enrolment in this study. Therefore, the influence of previous treatment and cancer status may lead to the different marker expression patterns from those in the healthy controls (**Supplementary Figure S2**). In the C/T alone group and C/T+IO group (**Figures 2A, E**), the expressions of CD45, CD3, CD8, and CD4 were not similar to those in the healthy controls and those in the IO alone group with obvious marker expression (**Supplementary Figure S2** and **Figure 2C**). In the IO alone group (**Figure 2C**), marked expressions of CD45, CD3, CD8, and CD4, and CD16 were found but the expressions of PD-1, TIM-3, and LAG-3 were not similar to those in the healthy controls with obvious marker expression. The different proportions of patients who received more than two lines of treatment among the C/T alone, IO alone, and C/T+IO alone group (**Supplementary Table S4**) may lead to the different intensity of marker expressions among these groups. In addition, part of the cell populations expressing CD45 in the C/T alone and C/T+IO group (**Figures 2A, E**) were clustered separately from the cell populations expressing CD3. There are several potential reasons for their partial separation in opt-SNE plots. First, the intensity and variance of CD45 expression across different cell types after treatment may be higher compared with CD3, leading to their partial separation in dimensionality reduction mappings. Second, CD45 has a large extracellular domain leading to epitope spreading across leukocytes and this high dimensionality in CD45 staining may separate it out from CD3. Third, biological heterogeneity in the cell-activation states, bound ligands etc. may alter CD45, and CD3 expression and lead to their partial separation (51, 59, 60). The C/T alone group exhibited markedly low numbers of PD-1^+^ cells, TIM-3^+^ cells, and LAG-3^+^ cells (**Figures 2A, B**). The overlay histograms further illustrated that the numbers of PD-1^+^ cells, TIM-3^+^ cells, and LAG-3^+^ cells in the C/T alone group increased after treatment and the number of PD-1^+^ cells in the IO alone group decreased after treatment (**Figures 2B, D**). To provide a comprehensive illustration of these findings, this study employed SPADE analysis to cluster and visualize distinct subpopulations of immune cells, including NK cells, CD8^+^ (cytotoxic) T cells, CD4^+^ T cells, B cells, and monocytes, in different treatment groups both before and after treatment (**Figures 3A, C, E**). The average percentages of various immune cell types among the total cells before and after treatment are depicted in **Figures 3B, D, F**, respectively. Notably, the proportions of NK cells were significantly higher after treatment than before treatment in the IO alone group (p < 0.001) and C/T+IO group (p < 0.01; **Figures 3D, F**). The proportion of CD4^+^ T cells was significantly higher after treatment than before treatment in the C/T+IO group (p < 0.001) (**Figure 3F**). However, the proportions of different immune cells did not exhibit significant changes before and after chemotherapy (**Figure 3B**).

### 3.5 Immunophenotyping of different lymphocyte subpopulations before and after treatment

**Figure 4:**
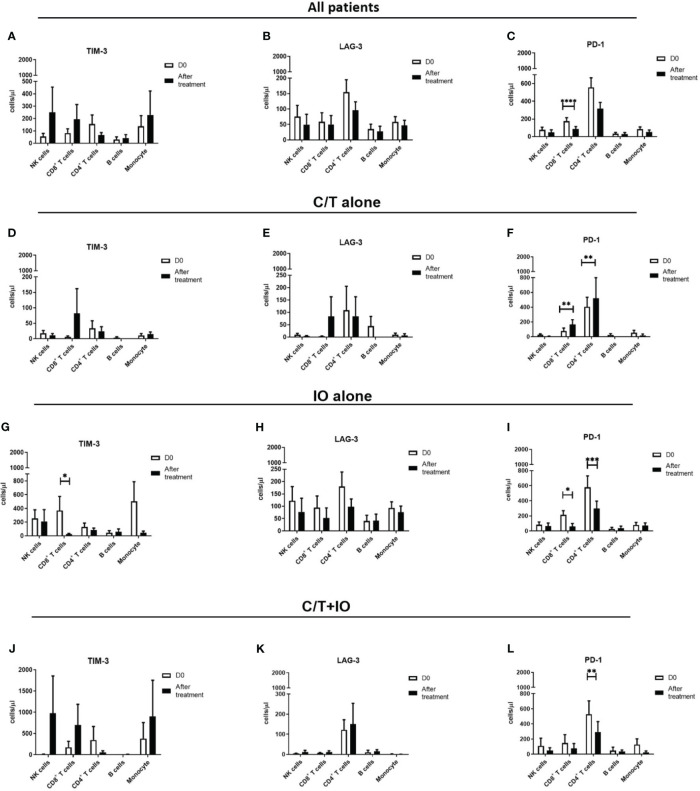
Circulating cell densities of lymphocyte subpopulation before and after treatment. The immunophenotyping analysis of different lymphocyte subpopulations which consisted of TIM-3^+^, LAG-3^+^, and PD-1^+^ cells before (Day 0, D0) and after different treatments revealed the cell counts per microliter of lymphocyte subpopulations for different treatment groups, including **(A-C)** all patients (****p < 0.0001), **(D-F)** chemotherapy (C/T) alone group (**p < 0.01), **(G-I)** immunotherapy (IO) alone group (*p < 0.05, ***p < 0.001), and **(J-L)** C/T+IO group (**p < 0.01) (n = 30).

**Figure 5:**
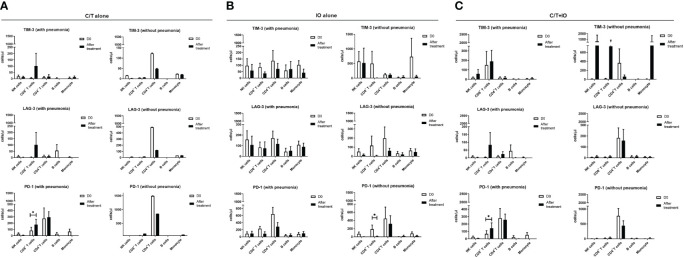
Circulating cell densities of lymphocyte subpopulation with or without pneumonia. The immunophenotyping analysis of different lymphocyte subpopulations which consisted of TIM-3^+^, LAG-3^+^, and PD-1^+^ cells before (Day 0, D0) and after different treatments revealed the cell counts per microliter of lymphocyte subpopulations for different treatment groups with or without pneumonia, including **(A)** chemotherapy (C/T) alone group (*p < 0.05), **(B)** immunotherapy (IO) alone group (*p < 0.05), and **(C)** C/T+IO group (*p < 0.05) (n = 27).

An immunophenotyping analysis of different lymphocyte subpopulations was conducted before and after various treatments (**Figure 4**). The cell counts per microliter of lymphocyte subpopulations for the study population and for each treatment group, namely C/T alone, IO alone, and C/T+IO, are presented in **Figures 4A–C, D–F, G–I, J–L**, respectively. With stratification by different exhausted T-cell subpopulations, the analysis revealed that PD-1^+^CD8^+^ (cytotoxic) T cells, and PD-1^+^CD4^+^ T cells (both p < 0.01; **Figure 4F**) significantly increased in the C/T alone group after treatment. However, in the IO alone group, TIM-3^+^ cytotoxic T cells (p < 0.05; **Figure 4G**), PD-1^+^CD8^+^ T cells (p < 0.05; **Figure 4I**) decreased after treatment. In both the IO alone and C/T+IO groups, PD-1^+^CD4^+^ T cells decreased after treatment (p < 0.001 and p < 0.01, respectively; **Figures 4I, L**). Subsequently, the cell counts per microliter of different lymphocyte subpopulations were assessed in the patients with and without pneumonia after treatment (**Figure 5**). The lymphocyte subpopulations in the C/T alone group, IO alone group, and C/T+IO group are presented in **Figures 5A–C**, respectively. In both C/T alone and C/T+IO groups, PD-1^+^CD8^+^ T cells significantly increased in patients with pneumonia after treatment (p < 0.05; **Figures 5A, C**). Nonetheless, in the IO alone group, PD-1^+^CD8^+^ T cells significantly decreased in the patients without pneumonia after treatment (p < 0.05; **Figure 5B**). Collectively, the findings indicate that the increased cell counts per microliter of PD-1^+^CD8^+^ T cells after treatment might be related to the occurrence of pneumonia following chemotherapy or immunotherapy. Survival analysis was further performed for patients with pneumonia and those without pneumonia in the prospective cohort. Patients were categorized into chemotherapy group and immunotherapy group which consisted of IO alone group and C/T+IO group according to their exposure to immunotherapy. Among all groups, there was no significant difference in OS between patients with pneumonia and those without pneumonia (p = 0.978; **Supplementary Figure S3**).

### 3.6 Cytokine profiles in different treatment groups

**Figure 6:**
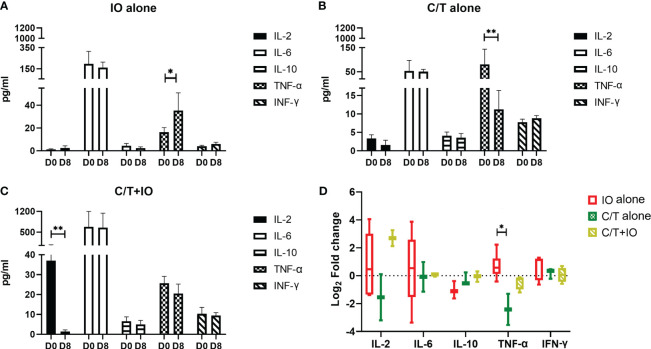
Cytokine profiles in different treatment groups. Circulating cytokine profiles consisting of the levels of IL-2, IL-6, IL-10, TNF-α, and IFN-γ were evaluated in different treatment groups before (Day 0, D0) and after treatment (Day 8, D8), including **(A)** immunotherapy (IO) alone group (*p < 0.05), **(B)** chemotherapy (C/T) alone group (**p < 0.01), and **(C)** C/T+IO group (**p < 0.01). **(D)** The changes of cytokine levels were revealed by using Log_2_ fold change in the concentration of different cytokines in Day 8 (D8) compared with that in Day 0 (D0) (*p < 0.05) (n = 30).

Cytokine profiles among different treatment groups were evaluated to elucidate the interplay between different treatments and the immune response. The levels of cytokines, including IL-2, IL-6, IL-10, TNF-α, and IFN-γ, were measured in peripheral blood specimens at baseline (Day 0) (D0) and after treatment (Day 8) (D8) through ELISA (**Figure 6**). Three treatment group showed different levels of cytokines observed at D0, indicating that there might be different immune-related characteristics among groups.

As presented in **Figure 6A**, TNF-α level significantly increased after IO alone treatment (p < 0.05) but TNF-α level significantly decreased after C/T alone treatment (p < 0.01; **Figure 6B**). IL-2 significantly decreased after C/T+IO treatment (p < 0.01; **Figure 6C**). The **Figure 6D** showed the change of cytokine levels by using Log_2_ fold change in the concentration of different cytokines in Day 8 (D8) compared with that in Day 0 (D0). TNF-α revealed higher level of Log_2_ fold change in the IO group than that in the C/T alone group (p < 0.05; **Figure 6D**). These findings suggest that IO alone, C/T alone, and C/T+IO treatment exert varying effects on the responses of TNF-α and IL-2.

## 4 Discussion

The advent of ICIs has led to considerable changes in the treatment landscape for NSCLC (10). However, the impact of immunotherapy on the risk of pulmonary infections following treatment remains unclear (21, 22, 61). The present study revealed that patients who received immunotherapy exhibited a lower risk of pneumonia and more favorable survival outcomes than those who received chemotherapy alone. CyTOF enabled quantification of circulating immune cells before and after treatment with immunotherapy and/or chemotherapy. Chemotherapy was found to increase the proportion of circulating exhausted T cells, particularly PD-1^+^CD8^+^ (cytotoxic) T cells. In contrast, immunotherapy led to a decrease in the proportions of circulating TIM-3^+^ and PD-1^+^ cytotoxic T cells and an increase in circulating NK cells. Patients who developed pneumonia after treatment exhibited higher numbers of circulating exhausted T cells, particularly PD-1^+^ cytotoxic T cells. Furthermore, the circulating TNF-α level significantly increased after treatment with IO alone but significantly decreased after C/T alone. These findings suggest that immunotherapy contributes to protective effects against pulmonary infections and enhances immune responses against pathogens by reducing the proportion of circulating exhausted T cells and increasing the levels of TNF-α and proportion of NK cells. The study findings also highlight the potential utility of exhausted T cells as circulating biomarkers to predict the risk of post-treatment infection in patients with advanced lung cancer.

The relationship between infection and immunotherapy has been a topic of debate (21, 22), with clinical trial data suggesting that immunotherapy does not increase the risk of infection, while real-world evidence has yielded inconsistent results (21, 36). In a retrospective cohort study, Malek et al. revealed that patients treated with immunotherapy had a lower risk of infection than those treated with chemotherapy (35). Our study yielded similar findings and provided a more comprehensive analysis of the proportion of circulating immune cells. The analysis revealed an increase in the proportion of NK cells following immunotherapy and an elevated proportion of exhausted T cells in patients receiving chemotherapy. NK cells have a crucial role in antibody-dependent cellular cytotoxicity against tumor cells. Traditionally, NK cells can defend against viral infections (62). Emerging evidences suggests that NK cells also contribute to the immune responses against bacterial infections, particularly in the upper and lower airways (63). As part of the innate immune system, NK cells are recruited to the lungs during infections, and they play a protective role (64). NK cells not only are cytotoxic but also produce proinflammatory cytokines such as TNF-α and IFN-γ (65). Marquardt et al. demonstrated that in humans, NK cells migrate between peripheral blood and lung tissue dynamically (63). Our findings indicated elevation of both circulating TNF-α levels and NK cells after IO treatment. The increase of NK cells in patients who received IO was associated with more favorable OS. Moreover, elevated peripheral NK cell activity has been linked to a reduced incidence of carcinoma (65, 66). It is reasonable to hypothesize that immunotherapy can modify the immune responses against pulmonary infections through changes in the NK cell numbers. Several clinical trials focused on NK cell therapies in NSCLC are underway (67), evaluating administration of either NK cells, cytokines, or antibodies. Future studies should comprehensively investigate the relationship between immunotherapy, NK cells, and the incidence of infection.

Our study indicated that chemotherapy induced more exhausted T cells, whereas fewer exhausted T cells were observed in the IO and C/T+IO groups. T-cell exhaustion plays a vital role in both cancer development and chronic viral infection (61). The loss of T-cell functions involves a series of changes, including reduced production of IL-2 and impaired secretion of TNF-α and IFN-γ (61). Reversing T-cell exhaustion may affect not only cancer treatment but also the risk of infection (30). Considering the complexity of host immunity and infection, different pathogens may lead to different outcomes (21). The PD-1/PD-L1 pathway has been suggested to increase the risk of tuberculosis reactivation (21). Tezera LB et al. observed that excessive TNF-α secretion could expedite mycobacteria growth (68). Although data on acute bacterial infection are limited, it has been hypothesized that blocking PD-1 in infectious diseases may be beneficial. Lazar-Molnar et al. demonstrated that PD-1 deficiency is associated with an increased bacterial load in the lungs (69). Our results indicate a dynamic clinical correlation between host T-cell exhaustion induced by chemotherapy and the opposite effects exerted by immunotherapy. Patients with pneumonia exhibited elevated numbers of PD-1^+^ cytotoxic T cells after C/T alone and C/T+IO treatment. However, those without pneumonia exhibited lower numbers of PD-1^+^ cytotoxic T cells after treatment with IO. The elevated numbers of PD-1^+^ cytotoxic T cells in the stroma are associated with worse outcomes of IO (12, 70). Kagamu et al. also revealed that immune signatures in peripheral blood could also predict treatment outcomes (42). Our findings show that PD-1^+^CD4^+^ T cells increased in C/T alone group after treatment but decreased in IO alone group and C/T+IO group after treatment. CD4^+^ T cells (helper T cells) are important in achieving the effective immune response to pathogens and clearance of infections through an integral role in the development and activation of CD8^+^ T cells and B cells (71, 72). PD-1 signaling has been reported to limit the accumulation of CD4^+^ T-cell in response to immunogenic stimuli (73). High expression of PD-1 on CD4^+^ T cells has been associated with poor clinical outcome in NSCLC (74). Previous studies demonstrated the expression of PD-1 upon activation on CD8^+^ and CD4^+^ T cells, NK T cells, B cells, and monocytes. The ligation of PD-1 appears to elicit inhibitory signals that dampen T-cell receptor signaling (75, 76), implicating that high expression of PD-1 which is a co-inhibitory molecule in T cells results in an immunosuppressed status and less function for anti-tumor response leading to progression of tumor and poor outcomes (73, 74, 77). Taken together, our results suggest that dynamic changes in peripheral blood immune signatures may serve as predictive markers of infection in patients with NSCLC undergoing cancer treatment.

The relationship between infection and immunotherapy in patients with cancer is difficult to clarify due to several challenges. First, the use of steroids to manage irAEs may increase the risk of infection (25). A retrospective cohort study of patients with melanoma who received immunotherapy found the use of steroids to be a risk factor for infection (25). A history of steroid use is an independent risk factor for infection and poor survival outcomes in patients receiving chemotherapy, immunotherapy, or both (35, 78, 79). Therefore, in this study, we identified steroid use as a risk factor and corrected for it in our multivariate analysis. Second, distinguishing infections from irAEs, particularly in the cases with pulmonary infections, can be challenging in clinical settings. Pneumonia and immunotherapy-related pneumonitis can have overlapping clinical presentations (21). Our single-center study addressed this by conducting detailed medical chart reviews to accurately identify cases with infection. Immunotherapy-related pneumonitis is associated with expanded inflammatory T-cell subtypes in BAL specimens (80). However, the analysis of immune signatures in BAL samples was beyond the scope of the present study. Third, different pathogens and infection types may lead to different outcomes (21, 22). We focused on pulmonary infections including pneumonia and respiratory tract infection. Pulmonary infection accounts for the majority of all infections in patients with NSCLC (21, 38)., and our study revealed the similar results. These infections are associated with an increased risk of poor outcomes in patients with pneumonia. Fourth, the IO group is inherently heterogenous. To elucidate the effect of IO, we focused on PD-1/PD-L1 inhibitors rather than anti-CTLA-4 inhibitors. However, a systemic review demonstrated that the risk of infection varied between PD-1 and PD-L1 inhibitors (34). This highlights the complexity of the immune system response to different agents. Despite these challenges, our study still offered a peripheral blood broad view of dynamic immune signatures. Our CyTOF results revealed that changes in immune signatures during the first week after treatment may be associated with the risk of infection. Considering the complexity of immunoreaction, steroid exposure, and cancer status, “dysregulated immunity” caused by immunotherapy could contribute to some infections (21). This perspective emphasizes the dynamic, complex nature of immune responses, rather than just treatment type. Future paired analyses of the tumor microenvironment and peripheral dynamic immune signatures, including multiple immune checkpoints may provide a more comprehensive understanding of immune reactions in patients with lung cancer.

There are several limitations in our study. First, one of the two cohort is a retrospective study in a single tertiary medical center, so some relevant data of immune profiles including immunophenotyping analysis, any grades of immune-related adverse events, cumulative dosage of PD-1/PD-L1 inhibitors, subsequent treatment after immunotherapy failure, tumor mutational burden, and tumor infiltrated lymphocytes were unavailable. Second, the prospective cohort was relatively small with limited generalizability due to the nature of a single tertiary center study. Third, the prospective cohort utilized blood specimens collected from the day before systemic treatment and the eighth day after treatment. The monitoring time is not long in our study, so the optimal monitoring period for immunophenotyping analysis remains to be elucidated in the future research. Fourth, some patients received prior cancer treatment before enrolment and the influence of previous treatment may lead to the different expression patterns of CyTOF markers from those in the healthy controls. Fifth, the effect of steroid use on immune signatures was not within the scope of the current study design, so its effects on immune signatures were unavailable. Despite these limitations, our data still provided insight into dynamic immune signatures for predicting infection risk after immunotherapy in advanced NSCLC.

## 5 Conclusion

Our study demonstrated that the administration of immunotherapy and chemotherapy leads to dynamic changes in lymphocyte subpopulations. Patients who developed pneumonia after C/T alone treatment exhibited higher cell densities of circulating PD-1^+^ cytotoxic T cells. After immunotherapy, the proportion of circulating NK cells and the level of TNF-α increased, and the cell densities of PD-1^+^ cytotoxic T cells decreased in patients without pneumonia. This study revealed that immunotherapy may be associated with a lower risk of pneumonia and more favorable OS than chemotherapy. Our finding highlights the potential benefits of immunotherapy in reducing the susceptibility to pulmonary infections through decreased exhausted T cells and the increased NK cells and TNF-α. Furthermore, exhausted T cells, NK cells, and TNF-α might play crucial roles in the immune responses against infections and could serve as potential circulating biomarkers for predicting the risk of infections following treatment. Future research should optimize strategies to prevent and manage post-treatment infections in patients with NSCLC, thereby contributing to prolonged patient survival.

## Data availability statement

The raw data supporting the conclusions of this article will be made available by the authors, without undue reservation.

## Ethics statement

The studies involving humans were approved by Institutional Review Boards of Taipei Veterans General Hospital. The studies were conducted in accordance with the local legislation and institutional requirements. The participants provided their written informed consent to participate in this study.

## Author contributions

Y-HL: Conceptualization, Data curation, Formal analysis, Funding acquisition, Investigation, Methodology, Project administration, Resources, Software, Supervision, Validation, Visualization, Writing – original draft, Writing – review & editing. C-IS: Conceptualization, Data curation, Formal analysis, Funding acquisition, Investigation, Methodology, Project administration, Resources, Software, Supervision, Validation, Visualization, Writing – original draft, Writing – review & editing. C-LC: Data curation, Formal analysis, Investigation, Project administration, Writing – review & editing. H-CH: Data curation, Investigation, Methodology, Project administration, Validation, Writing – review & editing. Y-MC: Conceptualization, Data curation, Formal analysis, Funding acquisition, Investigation, Methodology, Project administration, Resources, Supervision, Validation, Visualization, Writing – original draft, Writing – review & editing.
