# Supplementary material for: Dynamic immune signatures of patients with advanced non–small-cell lung cancer for infection prediction after immunotherapy
Source: Front Immunol. 2024 Jan 26;15:1269253. doi: 10.3389/fimmu.2024.1269253 (PMC10853389; doi:10.3389/fimmu.2024.1269253)

Supplementary Material

# Supplementary Tables

# Supplementary Table S1. Infection types (n = 283)

| Infection types | All patients (283) | C/T alone (139) | IO alone (63) | C/T+IO (81) | P value |
| --- | --- | --- | --- | --- | --- |
| Pneumonia (%)^a,b^ | 102 (36.0) | 59 (42.4) | 20 (31.7) | 23 (28.4) | 0.083 |
| Urinary tract infection (%) | 4 (1.0) | 2 (1.4) | 1 (1.6) | 1 (1.2) | 1.000 |
| Bacteremia (%) | 6 (1.4) | 4 (2.9) | 1 (1.6) | 1 (1.2) | 0.868 |
| Skin and soft tissue (%) | 20 (4.8) | 6 (4.3) | 6 (9.5) | 8 (9.9) | 0.210 |
| Others (%)^c^ | 38 (9.1) | 11 (7.9) | 9 (14.3) | 18 (22.2) | 0.011 |

# ^a^One patient (0.4%) with coronavirus disease 2019 (COVID-19) pneumonia in the IO alone group.

^b^89 (87.3%) patients with bacterial pneumonia and 13 (12.7%) patients with viral pneumonia, including 1 patient with COVID-19 pneumonia.

# ^c^Others included intraabdominal infection, colitis, neutropenic fever and occult infection.

# Abbreviation: C/T: chemotherapy; IO: immunotherapy.

# Supplementary Table S2. Multivariate analysis of infection episodes (n = 283)

|  | Univariate analysis | | | Multivariate analysis | | |
| --- | --- | --- | --- | --- | --- | --- |
|  | OR | 95% CI | p value | OR | 95% CI | p value |
| Male | 1.73 | 1.05-2.87 | 0.033 | 1.79 | 1.00-3.20 | 0.050 |
| Age ≥70 | 0.75 | 0.46-1.23 | 0.261 |  |  |  |
| Smoking history | 1.25 | 0.77-2.01 | 0.368 | 0.87 | 0.50-1.53 | 0.634 |
| ECOG PS ≥ 2 | 0.53 | 0.22-1.26 | 0.150 | 0.45 | 0.18-1.15 | 0.095 |
| DM | 1.47 | 0.80-2.71 | 0.213 |  |  |  |
| COPD | 0.90 | 0.40-2.03 | 0.795 |  |  |  |
| CKD | 4.22 | 0.93-19.21 | 0.063 | 5.05 | 1.06-24.15 | 0.043 |
| Neutropenia | 1.28 | 0.55-2.99 | 0.562 | 1.21 | 0.50-2.91 | 0.673 |
| Steroid | 0.91 | 0.45-1.85 | 0.798 | 0.87 | 0.42-1.81 | 0.717 |
| Treatment lines ≥ 2 | 0.74 | 0.45-1.22 | 0.237 | 0.76 | 0.44-1.30 | 0.319 |
| Radiotherapy | 1.05 | 0.60-1.82 | 0.876 | 1.15 | 0.63-2.08 | 0.656 |
| With immunotherapy | 1.09 | 0.68-1.76 | 0.716 |  |  |  |
| Abbreviation: CI: confidence interval; CKD: chronic kidney disease; COPD: chronic obstructive pulmonary disease; DM: diabetes mellitus; ECOG PS: Eastern Cooperative Oncology Group performance status; OR: odds ratio. | | | | | | |

| **Supplementary Table S3. Multivariate analysis of COVID-19 pneumonia episodes (n = 283)** | | | | | | |
| --- | --- | --- | --- | --- | --- | --- |
|  | Univariate analysis | | | Multivariate analysis | | |
|  | OR | 95% CI | p value |  |  |  |
| Male | 0 | Not calculable | 0.995 |  |  |  |
| Age ≥ 70 | 0 | Not calculable | 0.997 |  |  |  |
| Smoking history | 0 | Not calculable | 0.996 |  |  |  |
| ECOG PS ≥ 2 | 0 | Not calculable | 0.999 |  |  |  |
| DM | 0 | Not calculable | 0.998 |  |  |  |
| COPD | 0 | Not calculable | 0.998 |  |  |  |
| CKD | 0 | Not calculable | 0.999 |  |  |  |
| Neutropenia | 0 | Not calculable | 0.998 |  |  |  |
| Steroid | 0 | Not calculable | 0.998 |  |  |  |
| Treatment lines ≥ 2 | 16154748 | 0-upper limit not estimated | 0.996 | 8576598 | 0-upper limit not estimated | 0.995 |
| Radiotherapy | 24111565 | 0-upper limit not estimated | 0.995 | 3403436 | 0-upper limit not estimated | 0.994 |
| With immunotherapy | 11297027 | 0-upper limit not estimated | 0.996 | 7591448 | 0-upper limit not estimated | 0.995 |
| Abbreviation: CI: confidence interval; CKD: chronic kidney disease; COPD: chronic obstructive pulmonary disease; DM: diabetes mellitus; ECOG PS: Eastern Cooperative Oncology Group performance status; OR: odds ratio. | | | | | | |

| **Supplementary Table S4. Patient characteristics of the prospective cohort (n = 30)** | | | | | |
| --- | --- | --- | --- | --- | --- |
| Characteristics  (Patient number) | All patients (30) | C/T alone (6) | IO alone (18) | C/T+IO  (6) | P value |
| Age (median, range) | 63 (33-89) | 65 (52-81) | 68 (44-89) | 62 (33-65) | 0.008 |
| Male (%) | 12 (40) | 2 (33.3) | 9 (50) | 1 (16.7) | 0.087 |
| Smoking (%) | 14 (46.7) | 2 (33.3) | 9 (50) | 3 (50) | 0.516 |
| ECOG PS ≥ 2 (%) | 2 (6.7) | 0 (0) | 2 (11.1) | 0 (0) | 0.392 |
| DM | 2 (6.7) | 1 (16.7) | 1 (5.6) | 0 (0) | 0.664 |
| COPD | 1 (3.3) | 0 (0) | 1 (5.6) | 0 (0) | 0.723 |
| CKD | 0 (0) | 0 (0) | 0 (0) | 0 (0) | 0.299 |
| Neutropenia | 3 (3.3) | 0 (0) | 2 (11.1) | 1 (16.7) | 0.712 |
| Steroid | 4 (13.3) | 0 (0) | 2 (11.1) | 2 (33.3) | 0.498 |
| Treatment lines ≥ 2 | 10 (33.3) | 2 (33.3) | 5 (27.8) | 3 (50) | 0.936 |
| Radiotherapy | 6 (20) | 0 (0) | 5 (27.8) | 1 (16.7) | 0.228 |
| Infection (%) | 15 (50) | 1 (16.7) | 9 (50) | 5 (83.3) | 0.339 |
| More than one infection (%) | 8 (26.7) | 1 (16.7) | 4 (22.2) | 3 (50) | 0.843 |
| Infection require hospitalization (%) | 15 (50) | 1 (16.7) | 9 (50) | 5 (83.3) | 0.339 |
| ICU admission due to infection (%) | 4 (13.3) | 0 (0) | 3 (16.7) | 1 (16.7) | 0.545 |
| Abbreviation: C/T: chemotherapy; CKD: chronic kidney disease; COPD: chronic obstructive pulmonary disease; DM: diabetes mellitus; ECOG PS: Eastern Cooperative Oncology Group performance status; ICU: intensive care unit; IO: immunotherapy. | | | | | |

| **Supplementary Table S5. Multivariate analysis of pneumonia episodes (n = 27)** | | | | | | |
| --- | --- | --- | --- | --- | --- | --- |
|  | Univariate analysis | | | Multivariate analysis | | |
|  | OR | 95% CI | p value | OR | 95% CI | p value |
| Male | 0.70 | 0.16-3.17 | 0.643 | 0.75 | 0.70-8.04 | 0.813 |
| Age ≥ 70 | 0.81 | 0.16-4.20 | 0.804 |  |  |  |
| Smoking history | 0.93 | 0.21-4.11 | 0.919 | 0.91 | 0.12-7.05 | 0.927 |
| ECOG PS ≥ 2 | 0 | Not calculable | 0.999 | 0 | Not calculable | 0.999 |
| DM | 0 | Not calculable | 0.999 |  |  |  |
| COPD | 0 | Not calculable | 0.999 |  |  |  |
| CKD | 3069402201.42 | 0-upper limit not estimated | 1 |  |  |  |
| Neutropenia | 3836752751.77 | 0-upper limit not estimated | 0.999 | 4967115318.87 | 0-upper limit not estimated | 0.999 |
| Steroid | 1.89 | 0.23-15.74 | 0.557 | 0.72 | 0.03-18.76 | 0.842 |
| Treatment lines ≥ 2 | 2.33 | 0.49-11.17 | 0.289 | 1.53 | 0.20-11.41 | 0.681 |
| Radiotherapy | 4.86 | 0.72-32.87 | 0.105 | 7.83 | 0.44-140.48 | 0.162 |
| With immunotherapy | 1.61 | 0.26-10.13 | 0.614 | 1.13 | 0.12-10.93 | 0.917 |
| Abbreviation: CI: confidence interval; CKD: chronic kidney disease; COPD: chronic obstructive pulmonary disease; DM: diabetes mellitus; ECOG PS: Eastern Cooperative Oncology Group performance status; OR: odds ratio. | | | | | | |

# Supplementary Figure

**Supplementary Figure S1.** **The strategy used in the experiments of mass cytometry (cytometry by time-of-flight [CyTOF])**

The experiment started with a careful design of the antibody/probe panel. This was followed by sample analyzing by CyTOF 2 mass cytometer, uploading the flow cytometry standard (FCS) file to the online FCS file-processing platforms, and finally data analysis.


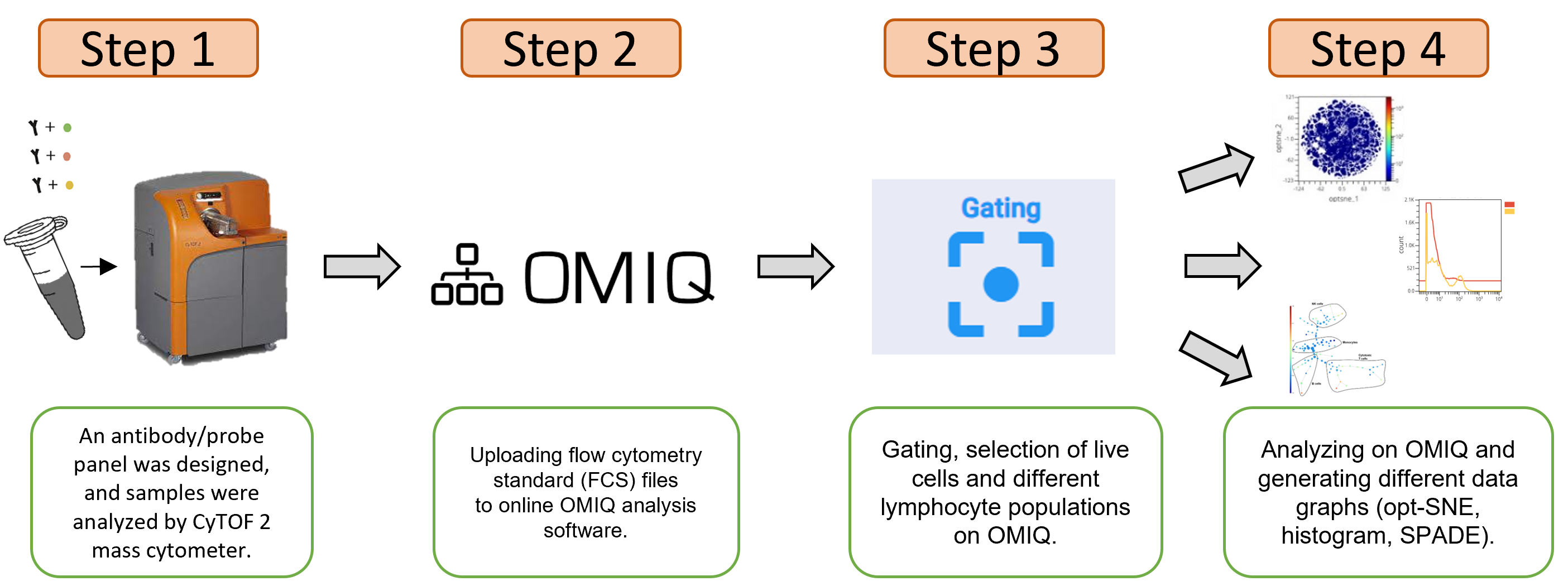


**Supplementary Figure S2. Immune signatures in the peripheral blood of patients from healthy controls, as analyzed through mass cytometry (CyTOF)**

The optimized t-Distributed Stochastic Neighbor Embedding (opt-SNE) plots (upper panels) and histograms (lower panels) depict the obvious expression of CyTOF markers, including CD45, CD3, CD8, CD4, CD16, PD-1, TIM-3, and LAG-3, in the peripheral blood mononuclear cells from healthy controls ( n = 3).


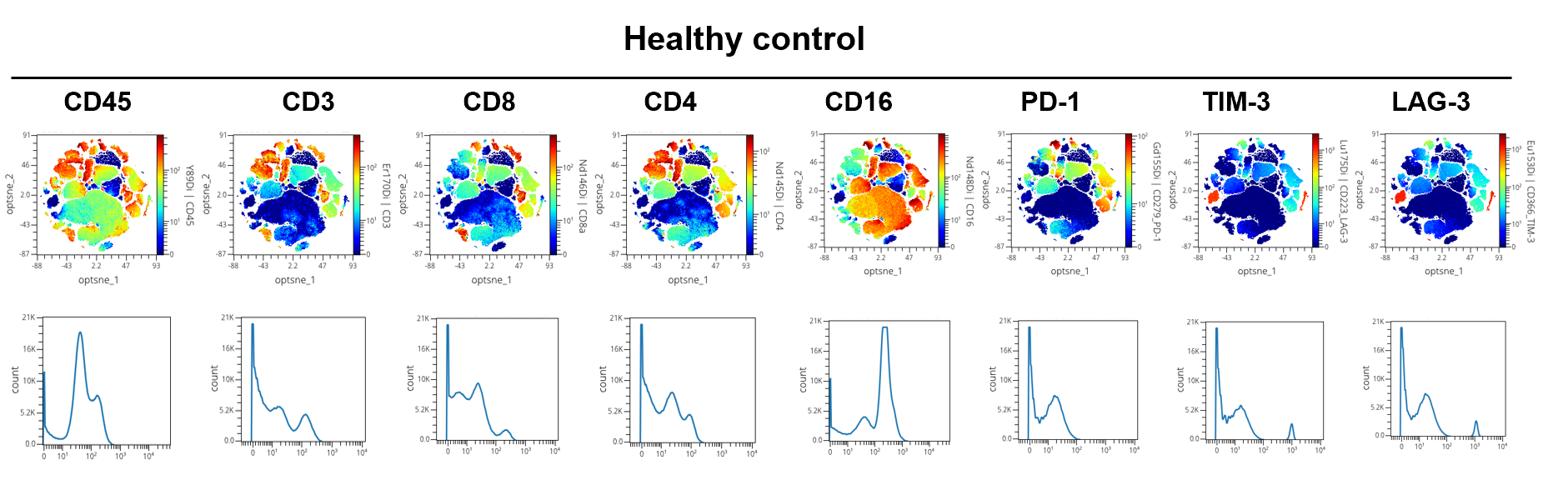


**Supplementary Figure S3.** **Survival outcomes between the chemotherapy and immunotherapy groups with or without pneumonia in the prospective cohort**

The patients were categorized into the chemotherapy group (C/T) and immunotherapy group (IO), and a survival analysis was performed for the patients with pneumonia or without pneumonia (n = 30).


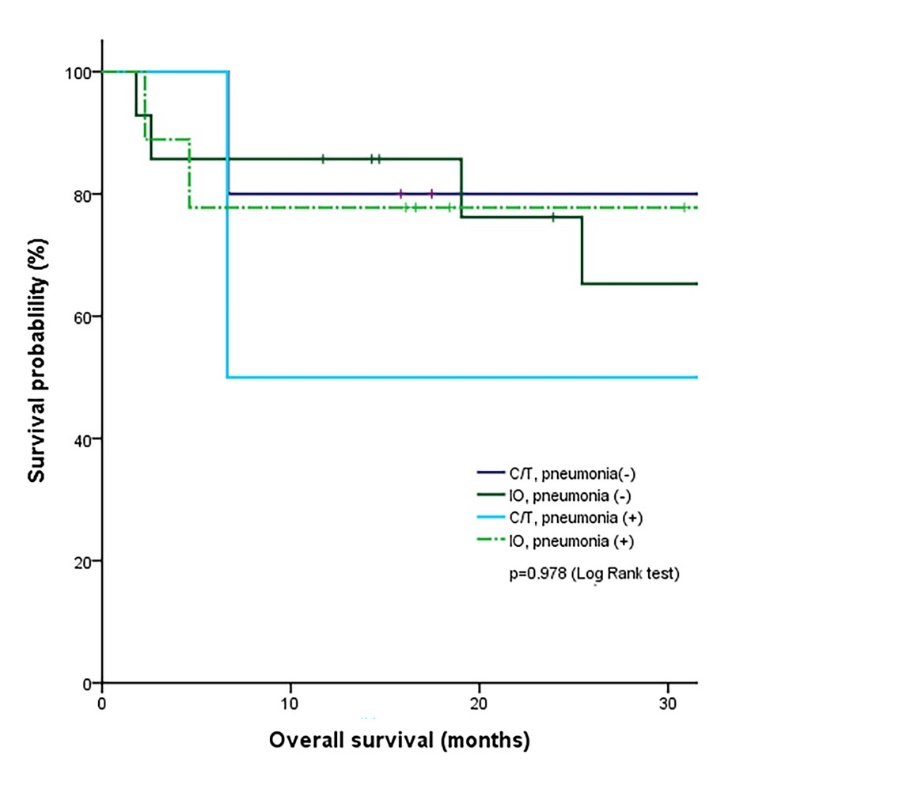

Supplement: Supplementary file 1 [file DataSheet_1.docx]
